# Characterization of microRNAs in Mud Crab *Scylla paramamosain* under *Vibrio parahaemolyticus* Infection

**DOI:** 10.1371/journal.pone.0073392

**Published:** 2013-08-30

**Authors:** Shengkang Li, Shuo Zhu, Chuanbiao Li, Zhao Zhang, Lizhen Zhou, Shijia Wang, Shuqi Wang, Yueling Zhang, Xiaobo Wen

**Affiliations:** 1 Guangdong Provincial Key Laboratory of Marine Biology, Marine Biology Institute, Shantou University, Shantou, China; 2 Department of Biology, College of Science, Shantou University, Shantou, China; Sun Yat-sen University, China

## Abstract

**Background:**

Infection of bacterial *Vibrio parahaemolyticus* is common in mud crab farms. However, the mechanisms of the crab’s response to pathogenic *V. parahaemolyticus* infection are not fully understood. MicroRNAs (miRNAs) are a class of small noncoding RNAs that function as regulators of gene expression and play essential roles in various biological processes. To understand the underlying mechanisms of the molecular immune response of the crab to the pathogens, high-throughput Illumina/Solexa deep sequencing technology was used to investigate the expression profiles of miRNAs in *S* *. paramamosain* under *V. parahaemolyticus* infection.

**Methodology/Principal Findings:**

Two mixed RNA pools of 7 tissues (intestine, heart, liver, gill, brain, muscle and blood) were obtained from *V. parahaemolyticus* infected crabs and the control groups, respectively. By aligning the sequencing data with known miRNAs, we characterized 421 miRNA families, and 133 conserved miRNA families in mud crab *S* *. paramamosain* were either identical or very similar to existing miRNAs in miRBase. Stem-loop qRT-PCRs were used to scan the expression levels of four randomly chosen differentially expressed miRNAs and tissue distribution. Eight novel potential miRNAs were confirmed by qRT-PCR analysis and the precursors of these novel miRNAs were verified by PCR amplification, cloning and sequencing in *S* *. paramamosain* . 161 miRNAs (106 of which up-regulated and 55 down-regulated) were significantly differentially expressed during the challenge and the potential targets of these differentially expressed miRNAs were predicted. Furthermore, we demonstrated evolutionary conservation of mud crab miRNAs in the animal evolution process.

**Conclusions/Significance:**

In this study, a large number of miRNAs were identified in *S* *. paramamosain* when challenged with *V. parahaemolyticus*, some of which were differentially expressed. The results show that miRNAs might play some important roles in regulating gene expression in mud crab under *V. parahaemolyticus* infection, providing a basis for further investigation of miRNA-modulating networks in innate immunity of mud crab.

## Introduction

In recent decades, rapid development of large-scale mud crab cultivation has been accompanied by the emergence of various diseases caused by bacteria, virus, and rickettsia-like organisms [1], which resulted in great economic losses in mud crab aquaculture. *V. parahaemolyticus*, a gram-negative bacterium, is one of the main pathogens of the brackish water-raised mud crabs in southeast China. When infected with *V. parahaemolyticus*, mud crab *S* *. paramamosain* showed the symptoms of lethargy, decreased appetite, reduced ingestion, and discolored carapace. The appendages of the diseased crab become pink, the ambulatory legs paralyzed, and the gills edematized. At the same time, the bacterial pathogen can be observed in tissues like blood, intestine, stomach, etc. [2–4]. It is vital and necessary to study the immune response of *S* *. paramamosain* to *V. parahaemolyticus* infection, whose underlying molecular mechanisms are not fully understood.

Recently, the non-coding miRNAs have been proved to regulate gene expression and function by repressing specific target genes at post-transcriptional levels in various biological processes via complementary binding to target mRNA’s 3'-UTR, leading to mRNA cleavage or protein translation blockage [5]. The first discovered miRNAs are lin-4 and let-7 in *Caenorhabditis elegans* as important regulators controlling the timing of larval development [6,7]. Till now, thousands of highly conserved miRNAs have been identified in different organisms, which function directly in the regulation of cell growth, differentiation, apoptosis, development, hormone secretion, and so on [8,9]. Moreover, miRNAs are currently estimated to comprise 1-5% of animal genes [10], controlling the activity of more than 60% of protein-coding genes [11].

Increasingly emerging evidences suggest that miRNAs play some important roles in the immune system in animals [12]. It is now evident that miRNAs have unique expression profiles in cells of the innate and adaptive immune systems. For example, 200 mature miRNAs have been detected in human acute monocytic leukemia cell line THP-1 under lipopolysaccharides (LPS) treatment [13]. A series of in vivo molecular tools have shown the critical role of miR-29 family in setting the molecular threshold for thymic production of T cells, T cell polarization and B cell oncogenic transformation in the adaptive immunity [14]. Also, miRNAs are being identified as key regulators in immune cell development and functions, as well as the defense against various foreign pathogens. The ligands of toll-like-receptor (TLR) 2, (TLR) 4, and (TLR) 5 significantly stimulate the expression of miR-146, leading to reduced expression of IRAK1 and TRAF6, which are important ingredients in the TLR pathway [15,16]. In prion-infected mouse brain tissues, miR-146a is over-expressed and may play a role as a potent modulator of microglial activation state [17]. During the innate immune response in mammals and invertebrates, the miR-155 is found to enhance the production of TNF-*a*, suggesting a positive role of miR-155 to regulate the release of inflammatory mediators [18–20]. Besides, miR-125b and miR-223 are also demonstrated to take an active part in the innate immune system [16].

Though much has been done for miRNAs identification and characterization in mammals and a few aquatic crustacean species [1,21–23], it keeps mostly unknown for miRNA transcriptome in mud crab *S* *. paramamosain* . Identification and isolation of immune-related miRNAs is the first step toward a thorough understanding of the roles of miRNAs in the innate immune system of mud crab.

In the present study, we examined the miRNA transcriptome from multiple tissues of mud crab *S* *. paramamosain* under the controlled and infected conditions with bacterial pathogen *V. parahaemolyticus* by using the high throughput Illumina/Solexa deep sequencing technology. By comparing mud crab miRNA transcriptome during *V. parahaemolyticus* infection and the control groups to known miRNAs in miRBase 19.0, we obtained 133 conserved miRNA families and 8 novel potential miRNAs in mud crab *S* *. paramamosain* were neither identical nor very similar to existing miRNAs. Subsequently, we detected and measured the expression levels of these miRNAs by stem-loop qRT-PCR assays and identified 161 significantly differentially expressed miRNAs (106 up-regulated and 55 down-regulated) during *V. parahaemolyticus* infection. These findings contribute to better understandings of miRNAs in regulating immune responses of mud crab to bacterial pathogen infection, and help for developing new strategies to prevent or treat *V. parahaemolyticus* infection in crustaceans.

## Results

### Overview of the high-throughput sequencing data

**Figure 1:**
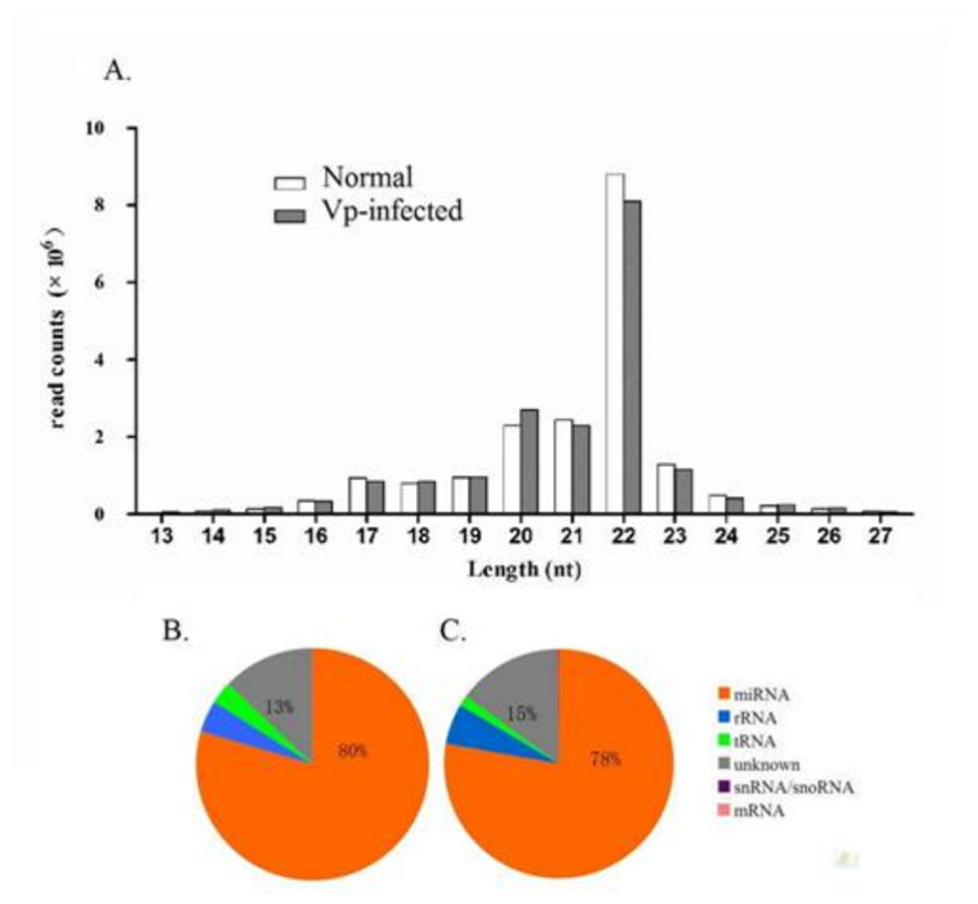
Length distribution, abundance and composition of small RNAs in mud crab *S* *. paramamosain* . (A) Length distribution and abundance of small RNAs in the control and infected groups. (B) Composition of small RNAs in the control group. (C) Composition of small RNAs in the infected group.

Two mixed RNA pools of 7 tissues (intestine, heart, liver, gill, brain, muscle and blood) from the *V. parahaemolyticus* infected mud crabs and the control groups, respectively, were subjected to high-throughput sequencing with the Illumina/Solexa platform. A total of 19,144,358 raw reads from the control group and 18,559,070 under the infected conditions, were obtained and uploaded to Gene Expression Omnibus (GEO: GSE39921). After removing some substandard reads, we analyzed the length distribution of the rest small RNAs (13–27 nt long) (control, 17,496,577, 91.90% of total, GEO: GSM981350; infected, 16,888,076, 91.51% of total, GEO: GSM981351) (Figure 1). The size distribution was similar between these two groups, and the most abundant small RNA was 22 nt long (Figure 1A). The small RNA sequences were compared with RNA databases (miRBase 19.0, http://www.mirbase.org, released at August 2012; Rfam, http://rfam.janelia.org; and Genbank, http://blast.ncbi.nlm.nih.gov). Reads from mRNA, rRNA, tRNA, snoRNA, and repeat sequences were removed. A quantity of 13,926,242 (^~^80%) and 13,138,560 (^~^78%) meaningful reads in the control and infected conditions, respectively, were remained for miRNA analysis (Figure 1 B, C).

### Identifying conserved miRNAs in *S. paramamosain*

To identify conserved miRNAs in mud crab, all small RNA sequences were Blastn searched against the currently known miRNAs and clustered on the basis of sequence similarity because some sequences probably came from the same precursor. To get a comprehensive knowledge of the conserved miRNAs in *S* *. paramamosain* , we compared mud crab miRNA transcriptome during *V. parahaemolyticus* infection and the control groups (Table S1) to known miRNAs in miRBase 19.0. Allowed one or two mismatches between sequences, after Blastn searches and further sequence analysis, total miRNAs in this two groups were belong to 421 miRNA families identified by miRBase (Table S2). Among the 421 families, 98 (23.3%) Spa-miRNA families were conserved in Arthropoda, 35 (8.3%) of which were conserved in Crustacea. Thus, 133 conserved miRNA families in mud crab *S* *. paramamosain* were either identical or very similar to existing miRNAs in miRBase, whereas the remaining 288 were known but non-conserved miRNAs in the crab miRNA transcriptome.

### Validation of miRNAs expression by stem-loop qRT-PCR

**Figure 2:**
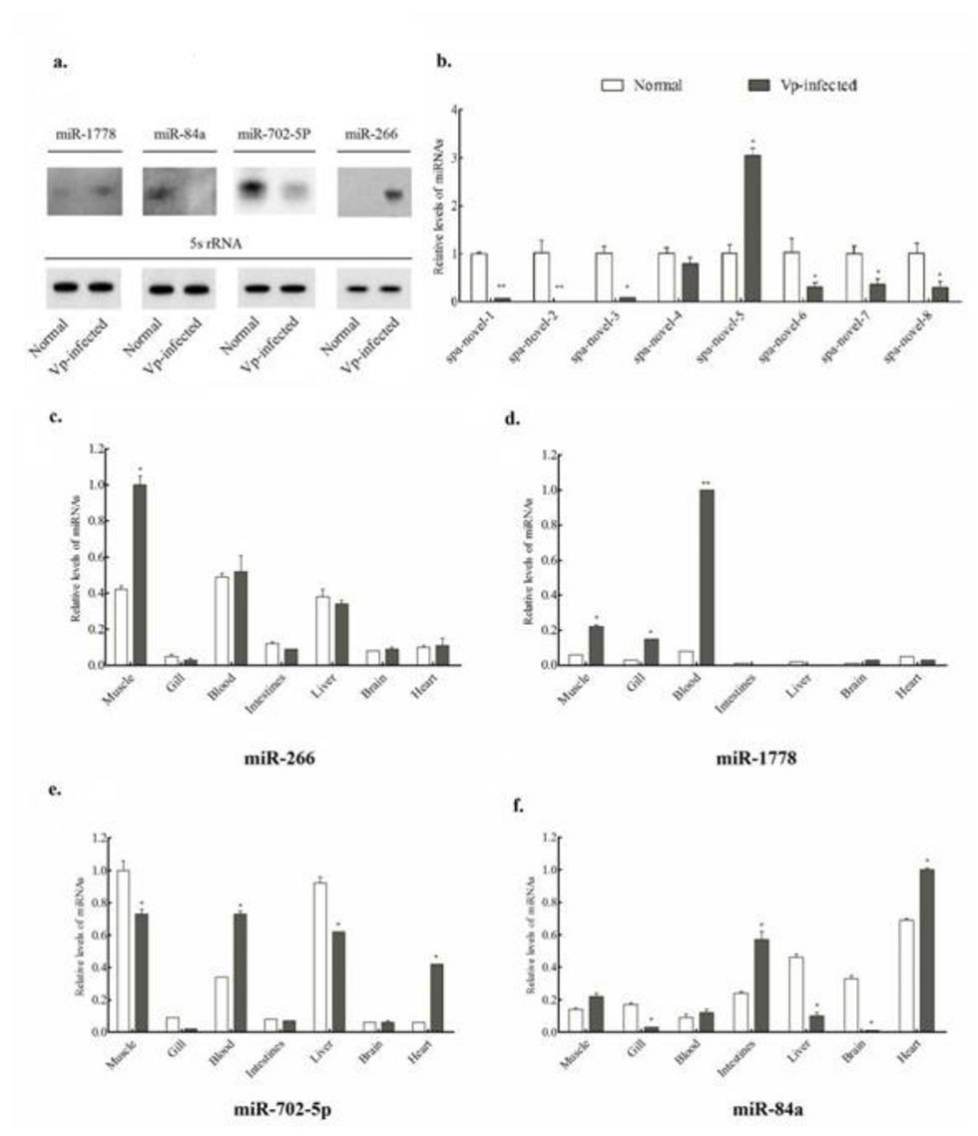
Expression profiles analysis of the conserved and novel potential miRNAs. (a) Validation of the four randomly-chosen differentially expressed miRNAs by Northern blotting. (b) Validation of eight novel potential miRNAs by qRT-PCR analysis. (c–f) The distribution of four randomly-chosen differentially expressed miRNAs (miR-266, miR-1778, miR-702-5p, miR-84a) in 7 different tissues (blood, intestine, heart, liver, gill, brain and muscle). Each reaction was repeated three times and the template amount was corrected by 5S rRNAs of mud crab. The significant difference of miRNA expression between the immune challenges and the control groups was indicated with asterisks (**: *P*<0.01, * *P*<0.05).

To validate the data acquired from sequencing, northern blotting analyses were conducted to determine the expression levels of 4 randomly-chosen significantly differentially expressed miRNAs (up-regulated: miR-266, miR-1778; down-regulated: miR-702-5p, miR-84a) with 5S rRNA gene of the mud crab as the internal reference. The results of the mixed RNA samples by northern blotting were well in accord with that of the sequencing by Solexa (Figure 2a), indicating the data presented here were in high quality. Furthermore, we measured the expression profiles of these four miRNAs by stem-loop quantitative real-time PCR assay in seven individual tissues. The data were subjected to analysis of one-way ANOVA followed by Student’s *t*-test (Figure 2c–f). miR-266 was expressed in all seven tissues tested in the treatment groups and the control groups with the highest expression level in the tissues of muscle, blood and liver. After *V. parahaemolyticus* infection, miR-266 increased its reads significantly in the muscle of the mud crab. Another up-regulated miR-1778, which had a very low reads in all tissues tested in the control groups, had a strong expression levels in blood and followed by that in muscle. As for two down-regulated miRNAs, miR-702-5p decreased its reads, most significantly, in muscle and liver; while miR-84a reduced its expression most in brain and gill after the *V. parahaemolyticus* infection in mud crabs. In summary, 4 differentially expressed miRNAs, all identified by Solexa sequencing, were validated by qRT-PCR assay, and the data showed a tissue specific characteristic only for miR-1778 (blood and muscle-specific).

**Figure 3:**
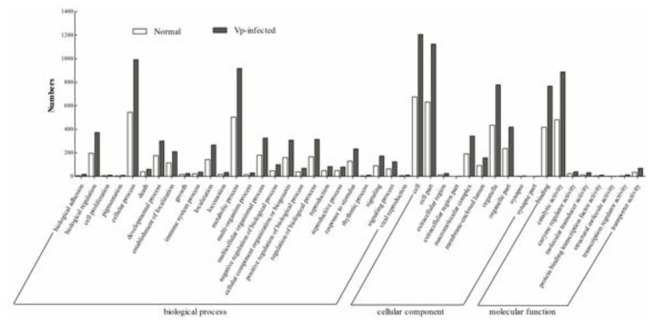
GO analysis of target genes in mud crab.

To better understand the function of the above novel as well as conserved mud crab miRNAs, putative targets of these miRNAs were predicted using the described criteria and methods [24]. All miRNAs were used for target analysis by search against *D* *. pulex* EST sequences and the annotated cDNA sequences of mud crab *S* *. paramamosain* (unpublished data). All miRNAs have multiple targets, and some even have more than 2000. Similarly, some *Unigenes* have varied miRNA target sites. Top 3 targets by the order of the scores were obtained for each miRNAs, and subjected to GO analysis to investigate gene ontology. The results demonstrated that the targets of miRNAs in mud crab could be involved in biological processes, cellular components and molecular functions (Figure 3).

### Identifying novel potential miRNAs in *S. paramamosain*

**Figure 4:**
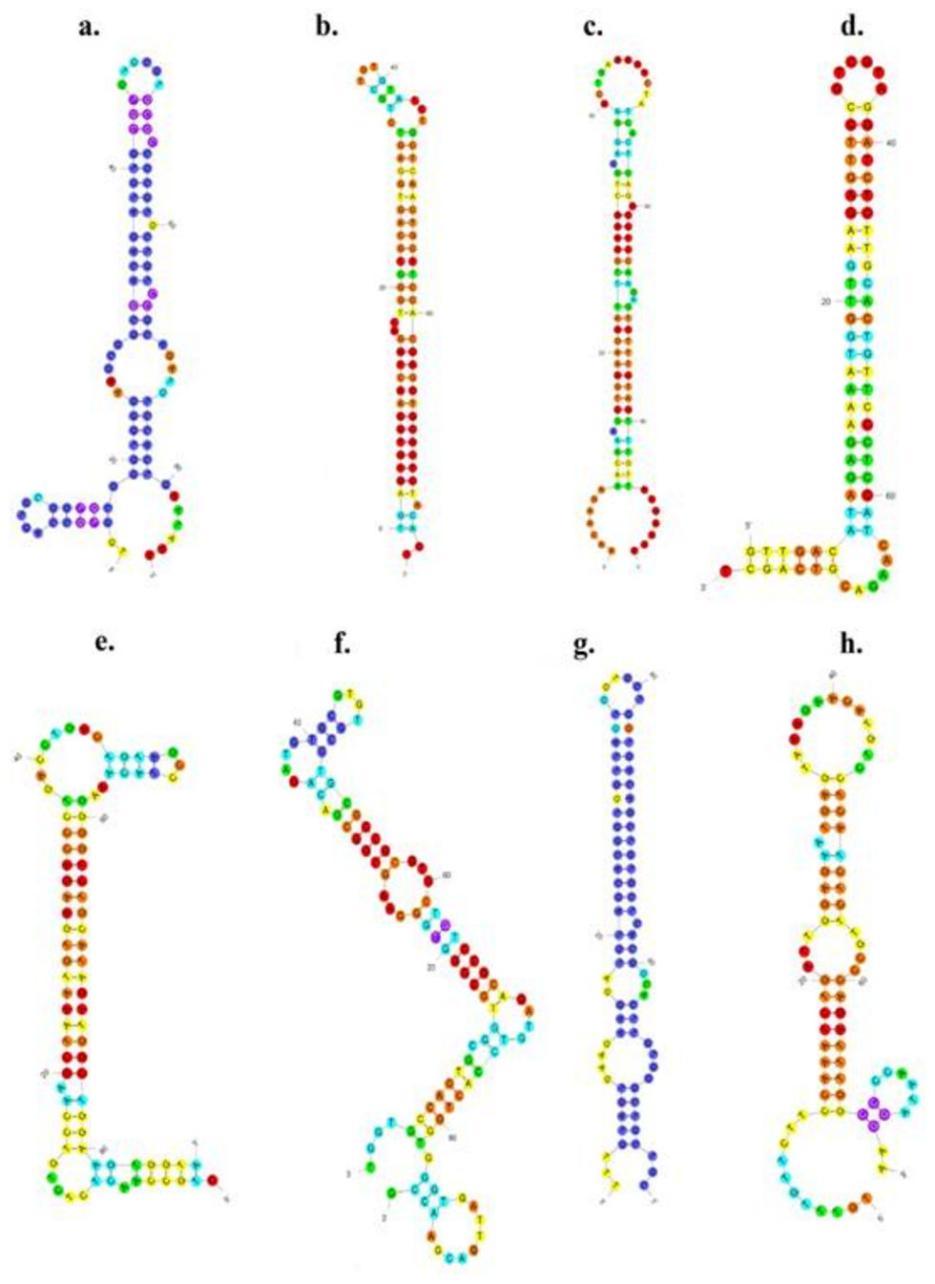
The folding secondary structure of putative precursor hairpins corresponding to eight novel potential miRNAs identified in this study. a-h stands for the secondary structure of precursor sequence of spa-novel-01, spa-novel-02, spa-novel-03, spa-novel-04, spa-novel-05, spa-novel-06, spa-novel-07, spa-novel-08, respectively.

Moreover, 8small RNAs were identified in the crab miRNA expressions of the infection group and the control group from precursors by using Mireap, designed by Beijing Genomics Institute (BGI), met the criteria established by Allen et al. [25] and were considered as putative novel *S* *. paramamosain* miRNAs (Table 1). Since the whole genomic sequence of mud crab is unavailable, we rely on the genome sequences of crustacean *Daphnia* *pulex* and our previous mud crab transcriptom database (unpublished data) as references to predict mud crab new miRNAs. The precursor sequences of the 8 novel potential miRNAs were verified by PCR amplification with *S* *. paramamosain* genomic DNA as templates (Figure S1), cloned and sequenced with the length varying from 76 to 100 nt (Table 1). The secondary structures were then predicted by Mfold to be a typical hairpin (Figure 4). Further, the minimum free energy (Mfe) value of the five miRNAs was estimated to range from -41.9 to -20.82 kcal/mol (Table 1), which were consistent with the results from previous researches [26,27].

**Table 1:** The novel miRNAs identified by Mireap in mud crab *S* *. paramamosain* .

**Name**	**Express**		**Sequence**	**Precursor length (nt)**	**Precursor sequence**	**Mfe (kcal/mol)**
	**Normal**	**Vp-infected**				
spa-novel-01	12	0	GCGGTGCGGGTGGGAAGCGGCG	**86**	TGGTGCCAGTGCGGTGCGGGTGGGAAGCGGCGACAGATGTGCGTGTGCGTGCGCCGCGTGCTGTCCGCAGATGTCCACTGAATAAA	**-34.90**
spa-novel-02	20	0	ACTGGACTGGGTGCAGTGGAGTC	**78**	TGAGAGTGCAACTGGACTGGGTGCAGTGGAGTCTGCTGTGTATCTGCTCAACTGCTTCCACCAGCTGCACTCTACACT	**-35.40**
spa-novel-03	11	0	AAAGCTGGAAGGATTTGGAGGCT	**92**	AACAAGAAACAAAGCTGGAAGGATTTGGAGGCTGGAGGAACTGAAAAACTATCACTCAGACCTCCAAGAGTCTTTCTAGTTTGTTAGGAAGA	**--31.60**
spa-novel-04	11	37	AGAAAATGGTTGAAGAGTTCCG	**75**	GTTGACATAGAGAAAATGGTTGAAGAGTTCCGTCGTAGGATCTCTTGCACTGTTCCCTCCATCAAGACGTCAGCT	**-21.80**
spa-novel-05	7	31	AGGGGTCTGCATAGATGGCT	**87**	CTGCCAACTCTCTGTCCAAGCTAAATGTGAAGACCCTGACCTGACTGTAGCCTACAAAGGGGTCTGCATAGATGGCTGGAAGTGGTA	**-27.80**
spa-novel-06	12	0	GCGGTGCGGGTGGGAAGCGGCG	**99**	TGGTGCCAGTGCGGTGCGGGTGGGAAGCGGCGACAGATGTGCGTGTGCGTGCGCCGCGTGCTGTCCGCAGATGTCCACTGGTGGGTGATTGACGAACCG	**-41.90**
spa-novel-07	5	10	AGAAGGATGTAGCACGGTGG	**78**	TTTGTAGTGAAGAAGGATGTAGCACGGTGGTTAGTGGTGCTGGTGACAGCTGTGCTCACGGCACTTGTGGGCTGCTGC	**-26.80**
spa-novel-08	12	0	TCCAAATCTGATTGGAGAATGA	**80**	TGTTTGTTCTTCCAAATCTGATTGGAGAATGAGTAAAGAACATGTCCTCATCTCTTGCCCAGATTTGGCCCCAATAGGAA	**-20.82**

The expression profiles of the 8 novel miRNAs during the pathogen infection groups and the control groups were assayed using qRT-PCR analysis and the signals were detected for all of them (Figure 2b). The stem-loop RT-PCR is a reliable method and a convenient way for detecting and identifying the novel miRNAs. In this study, we adopted this technique to validate and measure the expression of 8 novel potential miRNAs. All of these potential miRNAs were identified in *S* *. paramamosain* by Solexa sequencing. To aid in determination of *S* *. paramamosain* novel miRNA functions, we examined their expression in two libraries RNA pools. Of these 8 miRNAs, only Spa-novel-5 were significantly up-regulated, and the other 7 miRNAs were down-regulated, 6 of which were significantly reduced during the *V. parahaemolyticus* infection in *S* *. paramamosain* (Figure 2b). The data obtained here can form powerful evidence to support the existence of the novel miRNAs in *S* *. paramamosain* . The amplification plots and the melting plots of qRT-PCR analysis were provided as supporting information attached in Figure S2.

### Differential expression of miRNAs during *V. parahaemolyticus* infection

**Figure 5:**
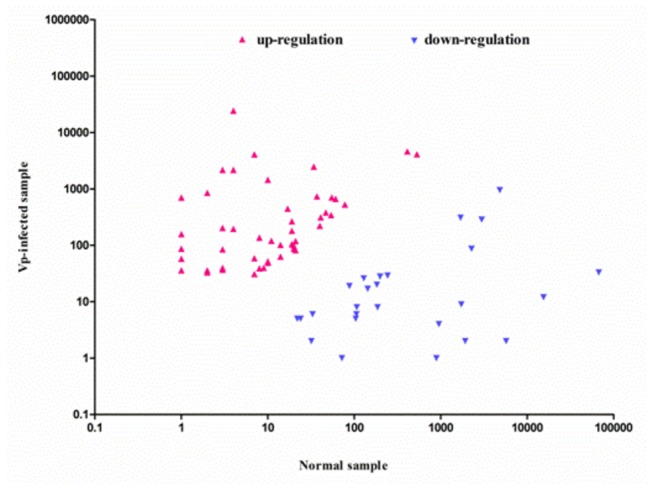
The differential expression of miRNAs between the infection treatment and the controls were showed by scatter plot. Each point in the figure represents a miRNA. Red points represent miRNAs with fold change > 2, blue points represent miRNAs with fold change ≤1/2.

To identify the infection-responsive miRNAs from *S* *. paramamosain* , the number of normalized miRNA reads of the treatment groups and the control groups were compared. Based on the sequencing results, the differential expression of miRNAs greater than two-fold were chosen for calculation. Of the differentially expressed miRNAs, 288 (48.6%) were expressed in both the control and infected mud crabs, whereas 196 (24.4%) and 151 (27.1%) were specifically expressed in the control and infected groups, respectively. After normalization, the expression levels of 161 miRNAs (161/421, 38%) (106 up-regulated and 55 down-regulated) were found to be significantly different between these two samples. The scatter plot for differentially expressed miRNAs between the treatment groups and the control groups was showed in Figure 5.

Three distinct miRNA families were found to change dramatically in the infection groups: miR-1, miR-2 and let-7. The miR-1 family, including miR-1, miR-1a, miR-1b, miR-1c, and miR-1*, had the highest total copy number of 18,356,231. The miR-2 family was the second most highly represented with 8 members including miR-2a-f, miR-2, miR-2b*, and miR-2f*. The let-7 family had 13 members including let-7, let-7a–k, and let-7f* (For more information, please see Table S1).

### Target prediction of differentially expressed miRNAs

The function of the differential expressed miRNA’s target is listed in Table S5. Actually, there are multiple targets for every miRNA. Meanwhile, for some genes, everyone has more than one miRNA binding sites. Part of some miRNAs targets would be immune related genes, such as miR-1345 targeting cryptocyanin and miR-135a* targeting kelch domain containing 2-like protein, Anoctamin-7 for miR-1224, calpain B for miR-1275, Immunoglubin I domain-containing protein for miR-412*, etc. Other predicted targets were annotated as transcriptor factor, such as transcriptase for miR-638, transcriptase-like protein for miR-1406 and transcription domain-associated protein for miR-198. Notably, several miRNAs targets would function in disease resistance, such as disease resistance protein (NBS-LRR) for two miRNAs miR328* and spa-novel-02. Due to the incomplete annotation of the transcriptomic data of *S* *. paramamosain* , the target of some miRNAs were function unkown, Moreover, The substantial part of the miRNAs identified during the infection in mud crab have no target genes at all. miRNA targets prediction and the function of the differential expressed miRNAs targets need further investigations.

### The phylogenetic evolution of miRNAs

**Figure 6:**
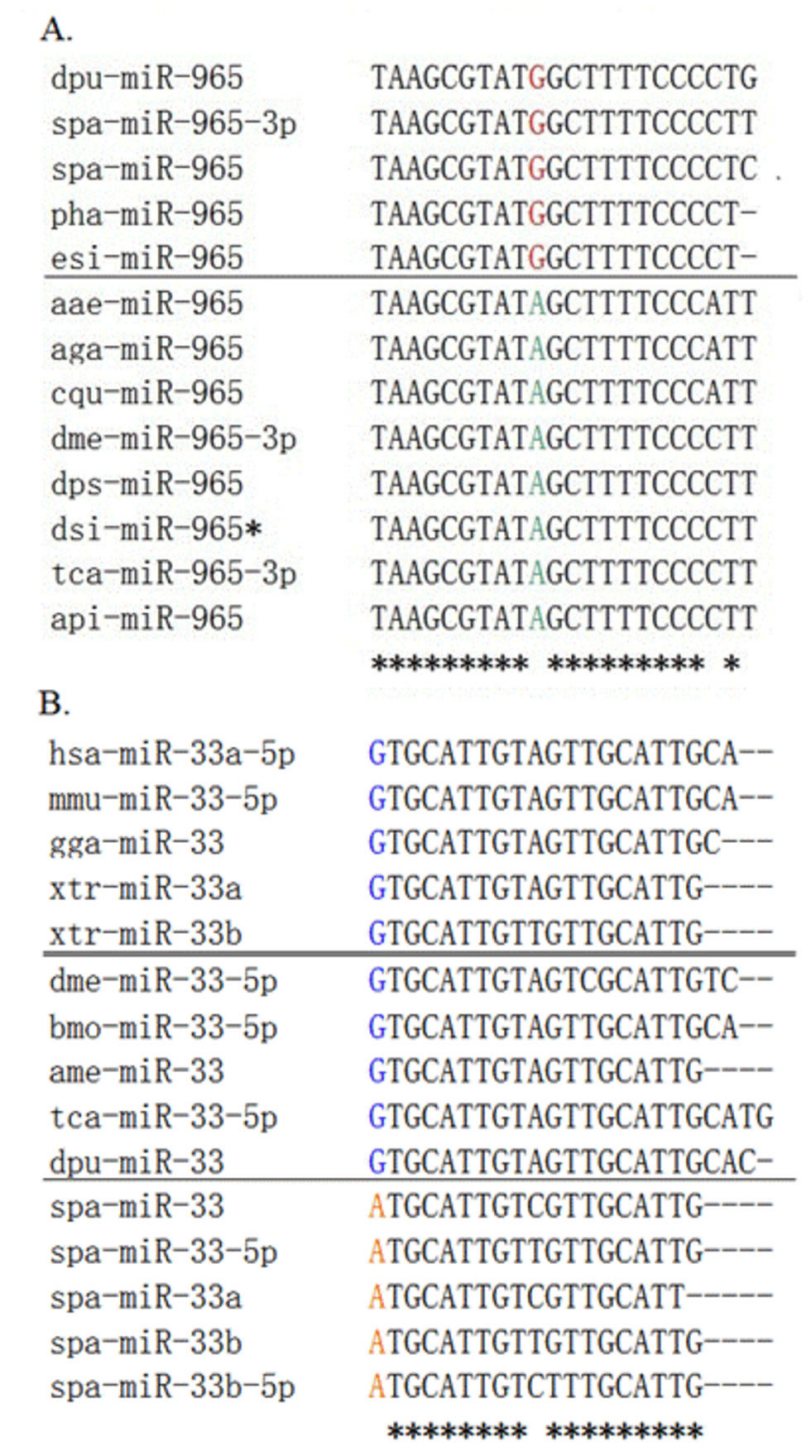
Phylogenetic evolution analysis of mir-965 (A) and mir-33 (B) in animals. (A) Nucleotides at the 10^th^ position of mir-965 are labeled in red in crustaceans, and in green in non-crustacean animals. (B) Nucleotides at the 1^st^ position of mir-33 are labeled in red in mud crab, and in blue in other animals. dpu: *Daphnia* *pulex* ; spa: *Scyllaparamamosain* ; pha: *Parhyalehawaiensis* ; esi: *Spiroplasma* *eriocheiris* ; aae: *Aedes aegypti*; aga: *anopheles gambiae*. cqu: *Culex* *quinquefasciatus* ; dme: *Drosophila melanogaster*; dps: *Drosophila* *pseudoobscura* ; dsi: *Drosophila* *simulans* ; tca: *Tribolium* *castaneum* ; api: *Acyrthosiphon pisum*; hsa: *Homo sapiens*; mmu: *Mus musculus*; gga: *Gallus gallus*; xtr: *Xenopus* *tropicalis* ; bmo: *Bombyx mori*; ame: *Apis mellifera*. Star line (“*"): consensus sequence.

Two conserved miRNAs (and respective homologoues), miR-965 (miR-965*, miR-965-3p) and miR-33 (miR-33a, miR-33b, miR-33-5p, miR-33a-5p, miR-33b-5p), were selected for further phylogenetic analysis (Figure 6). For miR-965 and its two homologoues, the seed regions, 1-9 and 11-19, were identical in mud crab and other crustaceans ( *D* *. pulex* , shrimp *Parhyalehawaiensis* and mitten crab *Spiroplasma* *eriocheiris* ). However, at the 10^th^ position, a transition mutation of A to G was found in miR-965 of mud crab when compared to that of non-crustacean animals (Figure 6A). For miR-33 and its five homologoues, instead, a transition mutation of G to A at the 1^st^ position could be identified only in mud crab (Figure 6B). Therefore, nucleotide sequences of miRNAs of *S* *. paramamosain* not only show the similar evolutionary patterns within the crustaceans, but also possess mud crab-specific mutations.

## Discussion

Although miRNAs have been studied extensively in invertebrates in the past several years, no systematic research has been reported for mud crab, whose farming is one of the most economically important mariculture in southeast China. In this study, we utilized the high-throughput sequencing platform (Illumina Genome Analyzer) to identify mud crab miRNAs during *V. parahaemolyticus* infection. 133 conserved miRNAs families from 421 miRNA families were identified using miRNA transcriptome of mud crab with 19.14 million raw reads of the control and 18.55 million raw reads of *V. parahaemolyticus* infected groups, respectively.

A total of 117 conserved miRNA*s were discovered in mud crab with *V. parahaemolyticus* challenge (Table S3). The function and significance of miRNA*s have often been neglected in previous researches because these sequences were usually regarded as important only for maintaining precursor secondary structure [5]. But recent evidences show that miRNA*s have substantial influence on miRNA and 3’-UTR evolution [28]. The results in this study further suggest that miRNA* might play an important role in regulating gene expression during *V. parahaemolyticus* challenge in mud crab, which are consistent with current reports in hemocytes of fluke [26], Japanese flounder [29] and flies [28].

Phylogenetic evolution analysis of 2 randomly chosen miRNAs showed that the miRNAs of *S* *. paramamosain* were distributed in a wide range of taxa from nematodes and insects to vertebrates. Meanwhile, miR-965 in *S* *. paramamosain* (Figure 6A) shows a similar evolutionary pattern to that in crustaceans, with some distinct differences from other non-crustacean animals, suggesting a similar ancient origin in crustaceans and conservation of mud crab miRNAs in the animal evolution process [26]. Further, nucleotide substitutions (A to G) may cause diversity of miRNA in regulating gene expression during animal defense response [29]. We also compared the identities of mud crab miRNAs with those from other crustaceans, such as mitten crab *Eriocheir* *sinensis* [1], shrimp *Parhyalehawaiensis* [22], and water flea *D* *. pulex* (miRBase 18.0). A total of 600 mud crab miRNAs matched (>95%) with their counterparts (data not shown). The results indicate that mud crab miRNAs have high homologies to miRNAs from *E. sinensis*, *P* *. hawaiensis* , and *D* *. pulex* .

Previous studies show that isomiRs may function importantly in the animals [30,31]. Our analysis revealed a large number of isomiRs in many miRNA families. In total, a large portion of isomiRs were found in the libraries of the infected mud crabs and the controls, which is consistent with previous reports [32]. Most isomiRs show differences at their 5’- and/or 3’-ends, likely resulting from variations in the pre-miRNA secondary structures that lead to variable cleavage sites for Dicer or Drosha. An example can be seen in Table S4, showing sequenced isomiRs for miR-1, which indicate that the majority of crab miRNA nucleotide variants are resulted from post-transcriptional modifications [32]. The presence of isomiRs may have distinct functions and require further researches.

More and more evidences have shown that miRNAs also play important roles in the immunity and immune response to foreign pathogen invasions in addition to various physiological processes [23,33]. Many miRNAs are differentially expressed in shrimp *Marsupenaeus* *japonicus* upon WSSV infection [21]. In another report, 24 miRNAs are characterized to take great effects on shrimp’s innate immunity, including phagocytosis, apoptosis and the pro-phenoloxidase system [23]. Therefore, the 661 differentially expressed miRNAs identified in this study might take part in mud crab’s immune response against pathogen infection. Specifically, three miRNAs (miR-146, miR-132, and miR-155) are significantly up-regulated in response to LPS in human monocytic cells [34]. A detailed survey of miR-146 expression in response to various microbial components reveals that an increase in miR-146 levels is induced by certain members of the Toll receptor family [34]. MiR-125a and miR-125b constitutively activate the NF-κB pathway by targeting the tumor necrosis factor alpha-induced protein 3 [35]. In this study, miR-146 and miR-125 were found differentially expressed in *V. parahaemolyticus* infected crabs, indicating that they might play an important role in the innate immunity in mud crab.

The let-7 family miRNA plays a conserved function in heterochronic developmental regulation [36,37]. Also, let-7 can inhibit the translation of the antimicrobial peptide diptericin by binding to its 3'-UTR [38]. Moreover, the 14-like transmembrane proteins, involved in phenoloxidase system, are targets of miRNA let-7 in shrimp [23]. Many miRNAs (let-7k, let-7b-5p, let-7h, let-7a) from let-7 family are down-regulated when mud crab is challenged with *V. parahaemolyticus*, indicating that they might also play an important role in the phenoloxidase system [23]. In humans, miR-124-a, and miR-124* are identified to affect influenza virus replication and host gene regulation [39]. In consistent with these observations, the up-regulation of miR-124 and miR-196b in the present study suggests that they might function in cellular processes such as inflammation, signal transduction, and apoptosis in crab.

To assess and define a putative function for a miRNA in the mud crab, a further step of target identification is necessary. Currently, the most efficient tool available for this is the bioinformatics approach facilitated by the high degree of homology between miRNA and its target sequences [40]. Consistent with previous reports [23,41], most of these targets in mud crab were animal-specific transcription factors, immune-related genes and disease resistance proteins (Table S5) As the whole genomic sequence of mud crab is unavailable, we rely mainly on our previous mud crab transcriptom database (unpublished data) and genome sequences of *Daphnia* *pulex* as references to predict miRNA targets. Some of the targets genes were annotated as function unknown, and most of the differentially expressed miRNAs even had no target, suggesting our work for mud crab CDS sequences annotation and the function of the differential expressed miRNAs targets need further investigations.

In conclusion, Solexa sequencing provided an accurate and efficient approach for studying small RNAs in mud crab *S* *. paramamosain* , as well as other species. We discovered a large amount of miRNAs during the pathogen infection in mud crab. We further analyzed the evolutionary pathway of the mud crab miRNAs, indicating the significance of miRNAs in animal evolution. 161 differentially expressed miRNAs were found in mud crab with *V. parahaemolyticus* infection. Constricted by the lack of mud crab genome information, the prediction of the target genes of miRNAs in mud crab is very limited. The results provided the basis for future analysis of miRNA function and mechanisms in mud crab immune response to *V. parahaemolyticus* infection.

## Materials and Methods

### Ethics Statement

The mud crabs used in this study were taken from a local crab farm (Niutianyang, Shantou, Guangdong, China). No specific permits were required for the described field studies, as the sampling locations were not privately owned or protected in any way. Furthermore these field studies did not involve endangered or protected species. The animals were processed according to "the Regulations for the Administration of Affairs Concerning Experimental Animals" established by the Guangdong Provincial Department of Science and Technology on the Use and Care of Animals.

### *V. parahaemolyticus* and *S. paramamosain* sampling and the challenge experiment

Forty healthy mud crabs (100 ± 10 g in weight) were collected from Niutianyang area in Shantou, China. Crabs were acclimated for 3 days in 1 m^3^ tanks (ten crabs per tank) in conditions (salinity: 8‰; temperature: 28^o^C) similar to those of culture ponds from which the crabs were obtained. Seawater was changed twice a day. Crabs were then fed with shellfishes for one week before sampling. *V. parahaemolyticus* isolated from diseased crabs (from Shantou crab farms) were cultured in 2216E medium at 28^o^C. Twenty crabs were inoculated individually in the ventral hind legs with 100 µL of *V. parahaemolyticus* (2.4 × 10^7^ cells/mL), while the other twenty crabs were injected with 100 µL of saline (0.9%) as the control group. 4 hours after the injection of *V. parahaemolyticus*, crabs showed a frail situation of decreased appetite, reduced ingestion, paralyzed ambulatory legs, discolored carapace, and milky appendages. 20 crabs from each of the two groups were collected for RNA isolation 24 hours after the challenge. Haemocytes were collected from blood samples after mixing with anti-coagulant solution and centrifugation at 800 g at 4^o^C for 20 min. The haemocytes and six other dissected tissues (intestine, heart, liver, gill, brain and muscle) were immediately frozen in liquid nitrogen and then stored at -80^o^C prior to RNA extraction.

### Tissue sampling and RNA isolation

Total RNA was extracted using TRIzol® (Invitrogen, Carlsbad, CA) according to the manufacturer’s protocol. Genomic DNA was removed by RNase-free DNase I digestion to gain high quality RNA samples, which were confirmed through measuring the ratio of 260/280 nm and the RNA integrity number (RIN) value [42]. After quantification, equal amount of RNA samples (2 µg) from the 7 tissues were mixed before being used for small RNA library construction.

### Small RNA library construction and Solexa sequencing

Small RNA fractions (18-30 nt) of the two mixed RNA samples under normal and infected conditions were enriched by polyacrylamide gel electrophoresis, and ligated with adapters to both 5'- and 3'-termini. Ligation products (62-75 nt) were reversely transcribed and those cDNAs were used for deep sequencing by an Illumina/Solexa G1 sequencer.

### Sequencing data analysis

Sequences of 50 nt were obtained via the Illumina/Solexa sequencing platform of Beijing Genomics Institute (Shenzhen, China). After removing of adapters, low quality tags, and contaminants, clean sequences were gained for further study. By aligning these sequences with data in miRBase 18.0 website, Genbank website, and Rfam database, small RNAs were separated into six groups: miRNA, rRNA, tRNA, unknown sequences, snRNA/snoRNA and degradation fragments of mRNAs. A total of 19,144,358 raw reads from the control group and 18,559,070 under the infected conditions have been deposited in Gene Expression Omnibus (GEO; accession number GSE39921). After removing the substandard reads, 13,926,242 reads from the control group and 13,138,560 reads from the infected group have also been deposited with GEO accession number (control: GSM981350; infected: GSM981351).

Known miRNAs were elucidated by comparing genome sequences of *Daphnia* *pulex* and our previous mud crab transcriptom database (unpublished data). To determine conserved miRNA, the filtered sequences were initially used to search the miRNA database, miRBase 19.0 with BLASTN. Only matching sequences (0-2 mismatches) were considered as conserved Spa-miRNA. Small RNA sequences were aligned to our previous mud crab transcriptom database to obtain precursor sequences in order to identify novel miRNAs. Contexts of perfectly matched hits were extracted as ±150 bp. The Mireap program developed by the Beijing Genome Institute (BGI) was used to analyze structural features of miRNA precursors to identify novel miRNA candidates. The resulting structures were retained as novel potential miRNAs only if they met the criteria described by Allen et al. (2005) [25]. The secondary structures of filtered pre-miRNA sequences were checked using Mfold [43]. In each case, only the structure with the lowest-energy was selected for manual inspection.

The sequence reads of the two libraries were normalized to 1 million by the total number of sRNA reads in each sample. The calculation of the *p*-value for comparison of the miRNA expression between the two libraries was based on previously established methods [44]. Specifically, the log_2_ ratio formula was: log_2_ ratio = log_2_ (miRNA reads in infection treatment/miRNA reads in control).

The following *p*-value formulae were used:

$$P\left(x|y\right)={\left(\frac{N_{{}^{2}}}{N_{1}}\right)}^{y}\frac{\left(x+y\right)!}{x!y!{\left(1+\frac{N_{2}}{N_{1}}\right)}^{\left(x+y+1\right)}}\begin{array}{c} C\left(y\leq y_{\min}|x\right)=\sum_{y=0}^{y\leq y_{\min}}p\left(y|x\right)\\ D\left(y\geq y_{\max}|x\right)=\sum_{y\geq y_{\max}}^{\infty }p\left(y|x\right) \end{array}$$

Where *N1* is the total number of reads in the sequencing library of the control, *N2* is the total number of reads in the sequencing library of the infection treatment, *x* is the number of reads for an miRNA in the control library, and *y* is the number of reads for an miRNA in the infection treatment library. All calculations were performed on a BGI Bio-Cloud Computing platform (http://www.genomics.cn/en/navigation/show_navigation?nid=4143). Normalized miRNAs of <1 were filtered in both libraries.

The unknown sequences were utilized to forecast novel miRNA by Mireap software, which could be used to analyze miRNA precursor’s structural features and recognize novel miRNA candidates. The secondary structures of alternative pre-miRNA sequences were verified by Mfold software using a minimum free energy method.

### Amplification of the miRNA precursors

The assay to amplify miRNA precursors was conducted as previously described [45]. Briefly, we extracted genomic DNA from the muscle tissue of the mud crabs according to the manufacture’s protocol and designed primers for 20 of 57 duplex-like miRNA: miRNA* pairs with Primer Premier 5.0 (Premier Biosoft International, Palo Alto, CA, USA). PCR reactions were performed with the *pfu* enzyme. Corresponding fragments were amplified by PCR and the length of amplification products was detected on 3% agarose gels. Fragments between 60 and 100 nt in length were subcloned into pMD19-T vector (Takara Bio, Dalian, CA, China) for sequencing analysis.

### Stem-loop RT-qPCR

Stem-loop RT-qPCR was used to confirm 8 novel potential miRNAs. The tissue distribution of 4 randomly-chosen differentially expressed miRNAs (miR-266, miR-1778, miR-702-5p and miR-84a) were also investigated by stem-loop RT-qPCR. All designed primers had been reported by Chen et al. [46] and listed in Table S6. cDNA was reverse-transcribed by PrimerScript ^®^RT reagent Kit with gDNA Eraser (Perfect Real Time, Takara, Dalian, China) using a method previously described [47]. We performed quantitative real-time PCR using the SYBR^®^ *premix Ex Taq* ^TM^ (Takara, Dalian, China) on an ABI Prism 7300 Sequence Detection System (Applied Biosystems, Foster City, CA). The reaction for each sample was performed in triplicate. Negative PCR products (no cDNA template) were prepared to detect any possible contamination. The specificity of primer amplicons was tested by analysis of a melting curve. The crab 5S rRNA was amplified as a reference gene for normalization using 2^-ΔΔT^ method [41,48,49]. All data were given in terms of relative miRNA expression as means±SE. The data were subjected to analysis of one-way ANOVA followed by Student’s *t*-test using SPSS 11.0 software, and the *p*-values smaller than 0.05 were considered statistically significant.

### Northern blotting analysis

Northern blotting analyses were performed following the procedure as described in miRNA Northern blot user manual (Signosis, Inc., Sunnyvale, CA). Briefly, approximately 10µg of total RNA was loaded onto a 15% polyacrylamide urea denature gel in 0.5% TBE and run at 70V for about 45 min. After transferred in 0.5% TBE onto a nylon membrane at 70V for 60 min, RNA samples were fixed on the membrane by UV cross-linking. The membranes were hybridized with 10µl of biotin-labeled miRNA-1778, miRNA-84a, miRNA-702-5p and miRNA-266 probe overnight respectively. The membranes were then rinsed with 1 X hybridization washing buffer, and subsequently hybridized with 8 µl of the amplifier for two hours at 42 ^o^C. After washed with 1x hybridization washing buffer for 30 min at 42 ^o^C, the membranes were blocked with blocking buffer at room temperature for 30 min, then detected with Streptavidin-HRP. After washing three times with 1x Detection washing buffer, the membranes were overlaid with substrate A and substrate B for 5 min. The image was acquired using a FluorChem FC2 imager (Alpha Innotech Corp, USA). The RNA load was normalized by mud crab 5S rRNA.

### miRNA Target prediction

Previous studies have proved that all miRNAs fulfilling the function of post-transcriptional gene regulation by binding to the target mRNA sequences in one or more perfect or near-perfect complementary site (s), bring convenience to predict animal miRNA targets simply using gene-homology search. To explore the potential function of miRNAs with significantly differential expression during the treatment with *V. parahaemolyticus* challenge in mud crab, we used the newly identified 161 differentially expressed miRNAs (known and novel miRNAs) to search their putative targets against annotated mud crab transcriptome data and published sequences from other invertebrates with RNAhybrid [24].

## Supporting Information

Figure S1 **Electrophoretic analysis of PCR products amplified from mud crab genomic DNA.** The primer pairs designed on the basis of the predicted precursor sequences of the novel miRNAs in mud crab. For each miRNA amplification, the negative control is used.
(TIF)

Figure S2 **The amplification plots and the melting plots for 8 novel potential miRNAs.**
(TIF)

Table S1 **Mud crab miRNAs transcriptome during the infection and the normal conditions**.
(XLS)

Table S2 **421 miRNA families in mud crab Scylla paramamosain compared with miRNAs from other species.** 1, hsa: *Homo sapiens*; 2, mmu: *Mus musculus*; 3, gga: *Gallus gallus*; 4, xtr: *Xenopus* *tropicalis* ; 5, dre: *Danio rerio*; 6, dme: *Drosophila melanogaster*; 7, aga: *Anopheles gambiae*; 8, bmo: *Bombyx mori*; 9, ame: *Apis mellifera*; 10, spa: *Scyllaparamamosain* ; 11, dpu: *Daphnia* *pulex* ; 12, tca: *Tribolium* *castaneum* ; 13, cel: *Caenorhabditis elegans*.
(XLS)

Table S3 **Mud crab star miRNAs during the infection**.
(XLS)

Table S4 **Top 100 editing forms of miR-1**.
(XLS)

Table S5 **The predicted targets of the differentially expressed miRNAs**.
(XLS)

Table S6 **Primer sequences designed for RT-qPCR.**
(XLS)
