# Supplementary figures and images for: Characterization of microRNAs in Mud Crab Scylla paramamosain under Vibrio parahaemolyticus Infection
Source: PLoS One. 2013 Aug 30;8(8):e73392. doi: 10.1371/journal.pone.0073392 (PMC3758354; doi:10.1371/journal.pone.0073392)

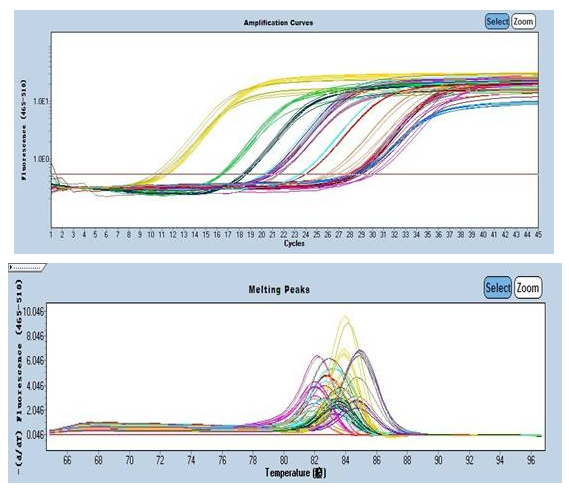

Supplement: Figure S2 — The amplification plots and the melting plots for 8 novel potential miRNAs. (TIF) [file pone.0073392.s002.tif]
